# MPDB 2.0: a large scale and integrated medicinal plant database of Bangladesh

**DOI:** 10.1186/s13104-021-05721-6

**Published:** 2021-08-06

**Authors:** Nazmul Hussain, Rony Chanda, Ruhshan Ahmed Abir, Mohsina Akter Mou, Md. Kamrul Hasan, M. Arif Ashraf

**Affiliations:** 1 Department of Biochemistry and Molecular Biology, Tejgaon College, National University of Bangladesh, Gazipur, 1704 Bangladesh; 2 Bio-Bio-1 Bioinformatics Research Foundation, Dhaka, Bangladesh; 3 Shahjalal University of Science and Technology, Sylhet, Bangladesh; 4 Biology department, University of Massachusetts Amherst, Amherst, MA USA

**Keywords:** MPDB2.0, Medicinal plant, Medicinal plant database of Bangladesh, Folk medicine

## Abstract

**Objective:**

MPDB 2.0 is built to be the continuation of MPDB 1.0, to serve as a more comprehensive data repertoire for Bangladeshi medicinal plants, and to provide a user-friendly interface for researchers, health practitioners, drug developers, and students who wish to study the various medicinal & nutritive plants scattered around Bangladesh and the underlying phytochemicals contributing to their efficacy in Bangladeshi folk medicine.

**Results:**

MPDB 2.0 database (https://www.medicinalplantbd.com/) comprises a collection of more than five hundred Bangladeshi medicinal plants, alongside a record of their corresponding scientific, family, and local names together with their utilized parts, information regarding ailments, active compounds, and PubMed ID of related publications. While medicinal plants are not limited to the borders of any country, Bangladesh and its Southeast Asian neighbors do boast a huge collection of potent medicinal plants with considerable folk-medicinal history compared to most other countries in the world. Development of MPDB 2.0 has been highly focused upon human diseases, albeit many of the plants indexed here can serve in developing biofuel (e.g.: *Jatropha curcas* used in biofuel) or bioremediation technologies (e.g.: *Amaranthus cruentus* helps to reduce cadmium level in soil) or nutritive diets (*Terminalia chebula* can be used in nutritive diets) or cosmetics (*Aloe vera* used in cosmetics), etc.

## Introduction

Since ancient times nature has always provided its favoured children (humans) with both remedies for fighting diseases and nutrition products to maintain good health via plants full of potent medicinal properties and nutritive values, [1] and our forefathers have utilized this boon from mother nature to the fullest, a sign of which can be still found in the local folk medicinal therapies [2–4]. While our predecessors hardly understood the underlying mechanisms and chemical mediators responsible for the efficacy of these plants, recent advancement in technology (which includes techniques, instrumentation, and automation in isolation and structural characterization) allows us to learn the underlying mechanisms of these herbal treatments in depth [5, 6] and discover the full potential for the usage of these plants full of potent medicinal properties and nutritive values [7, 8].

Several studies in recent times have suggested that plant active compounds or secondary metabolites possess immense potential for application in both research and clinical industries, for instance in developing nutraceuticals, nutrition products, biofuel technology, insecticides, flavouring agents, colouring agents, smelling agents, or fragrance, etc. [9–13]. According to records approximately 4,20,000 plant species are existing in nature among which about 10,000 to 15,000 plants have been studied for their medicinal and nutritional properties and about 200 of these plants or their active compounds have been adopted in western medicines [14, 15]. While natural substances are no longer used in modern medicinal therapies since the early decades of the last century, studies on bioactive molecules originating from plants continue to play a vital role in the development of novel medicinal therapies for emerging new diseases [16–18] as the parallel advancement of both biology and technology opens up an inexhaustible repertoire of naturally occurring compounds that have the potential to lead to the development of efficient and novel treatment strategies for diseases, both old and new [1, 7, 15].

**Fig. 1:**
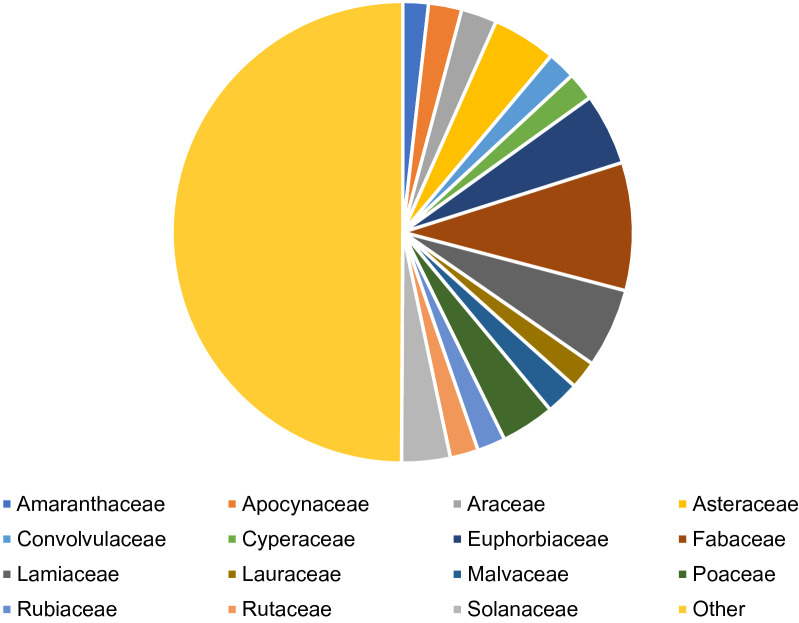
Pie chart demonstrates the most prominent plant families, expressed as percentage, from the MPDB 2.0. Families contain 10 or more than 10 plants are represented here individually, and all other families were listed as “other” category. Details numbers are provided in the Table 1

**Fig. 2:**
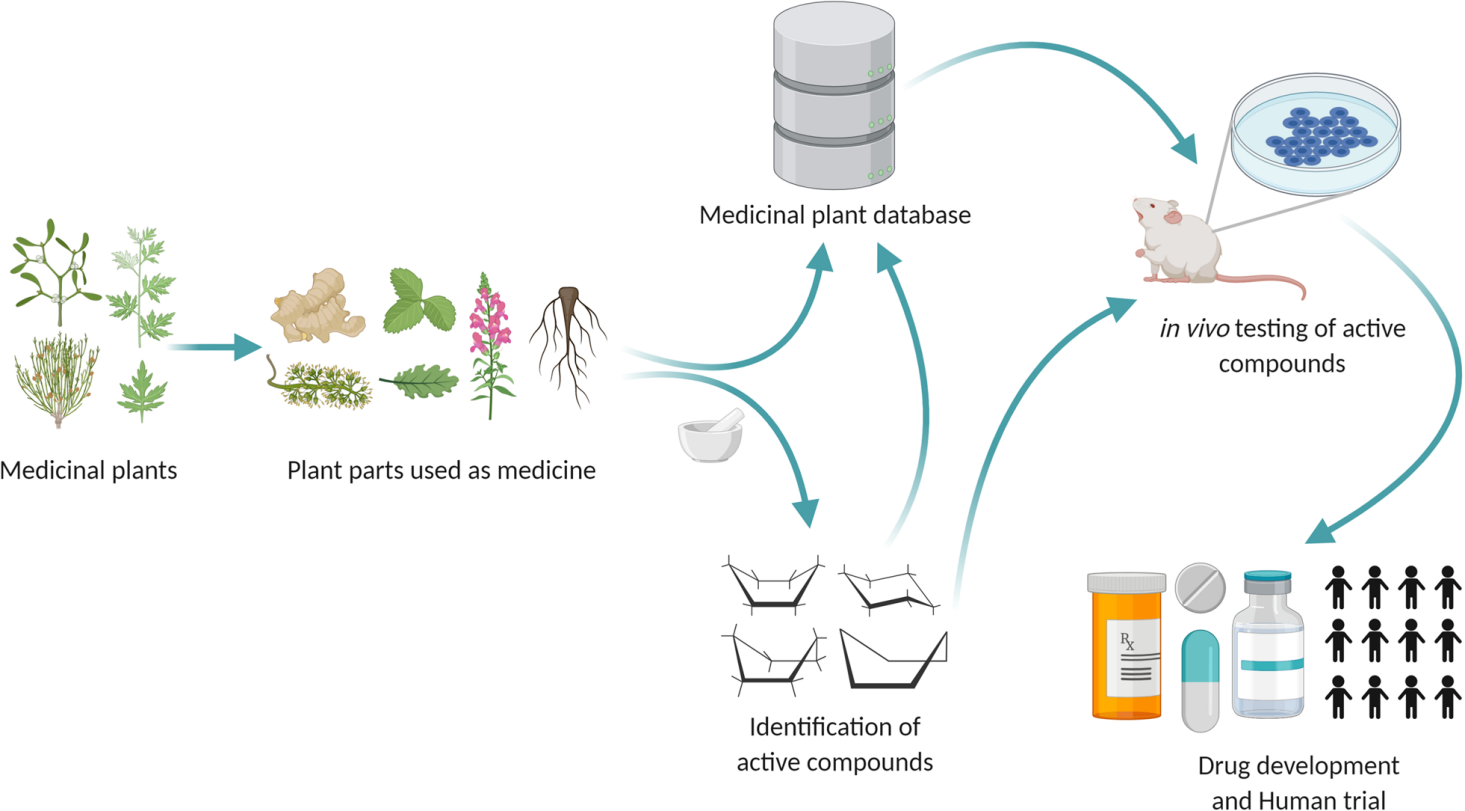
Involvement of medicinal plant databased such as MPDB1.0 and MPDB2.0 in drug discovery initiative

MPDB 2.0 database (plants of 122 family, 381 genus and 557 species), like MPDB 1.0 [19] is a user-friendly interface committed to rendering information (including their scientific names, family names, local names, utilized parts, related ailments, and active compounds) of plants around Bangladesh which have been suggested to have potent medicinal and/or nutritive properties, for both national and international researchers, health practitioners, drug developers and students who wish to study the medicinal and nutritional plants of Bangladesh. Interestingly, we have found that certain families are more prominent and appeared in a higher frequency in the collection of medicinal plant, MPDB 2.0. For instance, Asteraceae, Euphorbiaceae, Fabaceae, Lamiaceae, Poaceae, Solanaceae families are major source of medicinal plants in our database (Fig. 1; Table 1). To help the scientific community in advancing biological sciences the authors have dedicated the MPDB 2.0 database to be an open-source server. An updated medicinal plant database will enhance the scientific community to advance in the field of drug discovery (Fig. 2). In addition, we have created a promotional animated video (https://www.youtube.com/watch?v=tibx8MA6-Xs) for the MPDB 2.0 to reach wider audience and researchers.

**Table 1:** Major plant families in the MPDB2.0

Family	No of entries	% of each families
Amaranthaceae	10	1.795332
Apocynaceae	13	2.333932
Araceae	14	2.513465
Asteraceae	25	4.48833
Convolvulaceae	11	1.974865
Cyperaceae	11	1.974865
Euphorbiaceae	28	5.02693
Fabaceae	50	8.976661
Lamiaceae	31	5.56553
Lauraceae	11	1.974865
Malvaceae	13	2.333932
Poaceae	21	3.770197
Rubiaceae	11	1.974865
Rutaceae	11	1.974865
Solanaceae	19	3.411131
Other	278	49.91023
Total	557	100

## Main text

### Methods and materials

#### Literature mining

To collect the necessary plant data for the MPDB 2.0 database, a cluster of 43 literatures retaining information regarding medicinal and nutritive values along with respective folk therapies of Bangladeshi medicinal plants corresponded by both national and international research groups have been studied. The scientific names, family names, local names, utilized parts, and active compounds or secondary metabolites of the related plants have been included in the search parameters. To identify synonymous plant scientific names and reduce redundancies, The Plant List web server (http://www.theplantlist.org/) has been utilized.

#### Active compounds

For assembling literature retaining information of active compounds of the indexed Bangladeshi medicinal plants a separate search was conducted by two researchers independently and the PubMed web server (https://pubmed.ncbi.nlm.nih.gov/) has been exclusively utilized for this. The scientific names and scientific name synonyms have been implemented as search keywords.

#### Database preparation

VueJS, (https://vuejs.org/) a Javascript framework has been implemented for building the frontend of MPDB 2.0. ElasticSearch, (https://www.elastic.co/) a distributed storage and analytics engine has been utilized as the backend of this database. For ensuring the robustness of search functionality Edge-ngram tokenizer has been employed alongside ElasticSearch’s standard tokenizer when indexing the documents. FastAPI, (https://fastapi.tiangolo.com/) a Python web framework has been utilized in building the middleware for this database which translates the request-response between frontend and ElasticSearch.

#### Database access

**Fig. 3:**
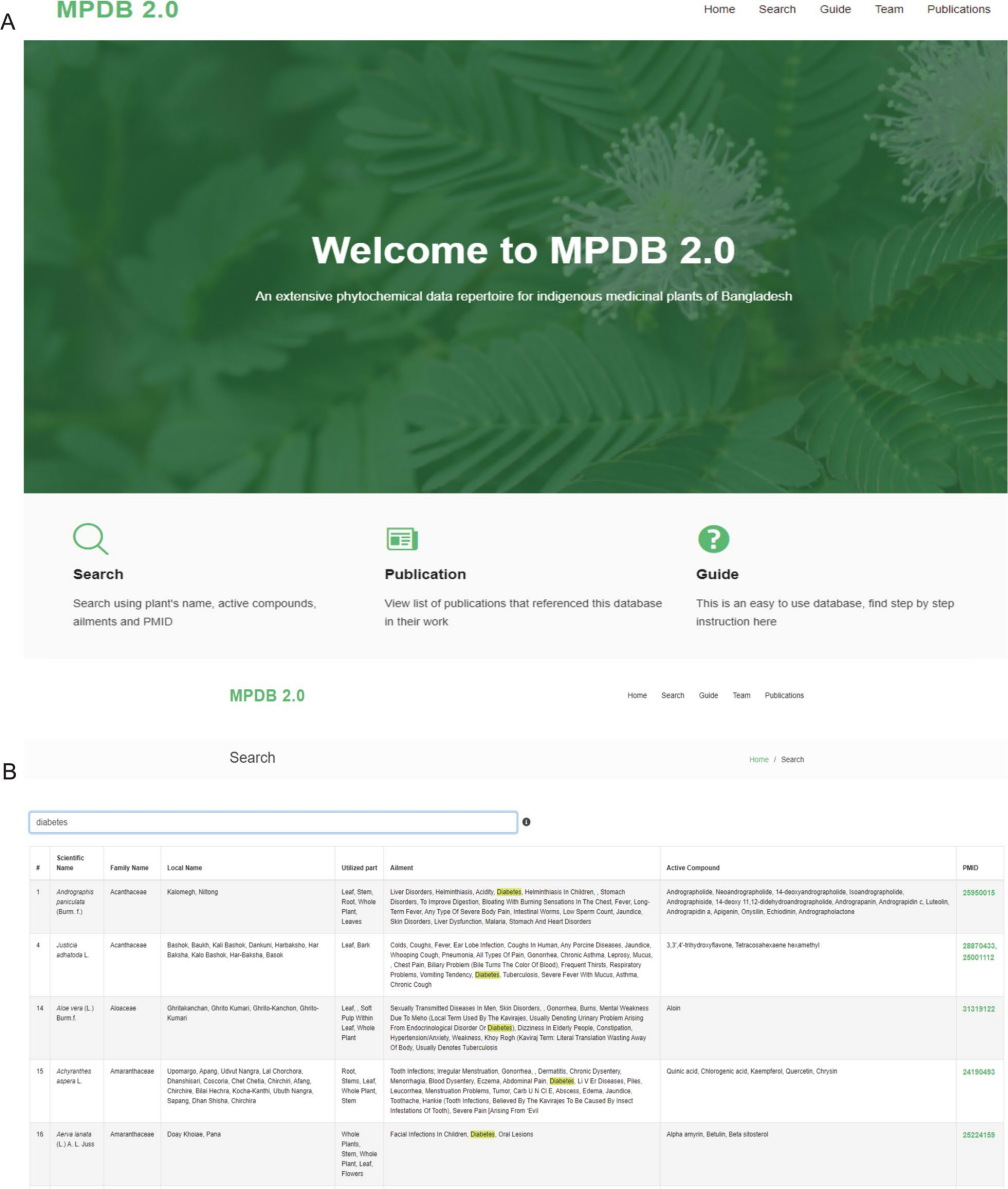
**A** Interface of MPDB2.0 database. **B** Keyword search result demonstrates related plant, their scientific names, source of plant, utilized parts, ailment, available active compounds, source of article, and hyperlinked PubMed ID of the reference papers

The MPDB 2.0 database contains five pages among which the “Search” (Fig. 3) page displays and gives access to the indexed Bangladeshi medicinal plants information along with their active compounds list. The search functionality within this page enables a user to query the database using one or more keywords (scientific name, local name, utilized parts, ailments, active compounds, and PMID) for normal or Boolean search. Every keyword entered is attempted to match with all the fields unless the field is specified. Several singles keyword queries matched with different fields are illustrated in Fig. 3. Users can search by stitching the keywords with AND/OR operator. Some plant entries contain multiple PMID entries as when collecting active compounds data, the authors used multiple references, in such cases clicking on the PMID section allows the users to see which active compounds belong to which publications, improving the overall user-friendliness of the database (Fig. 3).

### Conclusion

MPDB 2.0 is the continuation of MPDB 1.0, while it is established upon the same initial structure as MPDB 1.0, it contains a much larger data repository compared to its predecessor and the released version is much more refined and user friendly than MPDB 1.0. The MPDB 2.0 database has been dedicated to being a promising webserver engine for the initial screening of phytochemicals in silico drug development.

## Limitations

In order to reduce over-complexity, when some plants had multiple publications describing different active compounds composition, only the publication with the highest number of validated active compounds have been indexed in the database, which reduces the possible data density of the database. Also, the plant information indexed in MPDB 2.0 with the exceptions of active compounds and PubMed IDs have been collected from publications on Bangladeshi medicinal plants, since there has been a server lack of wet lab works on these Bangladeshi plants in Bangladesh, and their metabolite composition has not been evaluated locally. A separate literature mining was conducted to identify the active compounds of these plant species from publications and research works of other countries. Since the geodemographic distribution of plants has a considerable effect on the composition of their metabolites, the accuracy of the active compounds listed in the database remains open to questions. The authors hope to remove these inadequacies in future editions of the MPDB.

## Data Availability

Data used in this study or produced is either available from the public domain or mentioned here in the manuscript. Apart from that, authors always welcome to share the data required for reviewers and other researchers.

## Abbreviations

MPDB 2.0: Medicinal plant database 2.0
PMID: PubMed unique identifier
